# Current State of the Art and Potential for Construction and Demolition Waste Processing: A Scoping Review of Sensor-Based Quality Monitoring and Control for In- and Online Implementation in Production Processes

**DOI:** 10.3390/s25144401

**Published:** 2025-07-14

**Authors:** Lieve Göbbels, Alexander Feil, Karoline Raulf, Kathrin Greiff

**Affiliations:** Department of Anthropogenic Material Cycles, RWTH Aachen University, Wüllnerstraße 2, 52062 Aachen, Germany

**Keywords:** sensor-based, quality assurance, quality control, quality monitoring, quality prediction, waste processing, primary production, secondary production, recycling

## Abstract

Automated quality assurance is gaining popularity across application areas; however, automatization for monitoring and control of product quality in waste processing is still in its infancy. At the same time, research on this topic is scattered, limiting efficient implementation of already developed strategies and technologies across research and application areas. To this end, the current work describes a scoping review conducted to systematically map available sensor-based quality assurance technologies and research based on the PRISMA-ScR framework. Additionally, the current state of research and potential automatization strategies are described in the context of construction and demolition waste processing. The results show 31 different sensor types extracted from a collection of 364 works, which have varied popularity depending on the application. However, visual imaging and spectroscopy sensors in particular seem to be popular overall. Only five works describing quality control system implementation were found, of which three describe varying manufacturing applications. Most works found describe proof-of-concept quality prediction systems on a laboratory scale. Compared to other application areas, works regarding construction and demolition waste processing indicate that the area seems to be especially behind in terms of implementing visual imaging at higher technology readiness levels. Moreover, given the importance of reliable and detailed data on material quality to transform the construction sector into a sustainable one, future research on quality monitoring and control systems could therefore focus on the implementation on higher technology readiness levels and the inclusion of detailed descriptions on how these systems have been verified.

## 1. Introduction

As with primary material production chains, automated quality assurance is gaining popularity in the processing of secondary material streams [1]. In the case of waste processing, sensors have proven to be an essential technology when it comes to sorting [1,2,3], among other things, to provide pre-concentrates with high purity and thus ensure high-quality input for further process steps [4,5]. However, automatization for monitoring and control of product quality in processing streams is still in its infancy when it comes to waste processing [1,6]. For instance, in the area of construction and demolition waste (CDW) processing, the current state of the art for the analysis of mechanical properties of a processed material is manual sampling with a subsequent manual sieve analysis [7]. One possible reason for the lack of automatization for product quality monitoring and control is the general complexity of waste material streams, for instance, in terms of material heterogeneity [2,7,8,9], occlusion of particles [7,10,11,12], rough environments [7,9], and profitability [9,13]. In particular, intricacies, such as the high throughput of bulk material streams with high particle size and shape variations, large vibrations present in and around processing machines, large amounts of dust, and general weather influences such as heavy rain and sunshine, hinder straightforward implementation of sensor technology or advanced recycling in a more general sense [6,14]. Moreover, the high share of invisible particles due to bulk forming, together with high variances in the material stream composition and the wide variety of material types even within a single plant, complicates the processing of sensor data and subsequent determination or close approximation of the material quality [15]. So, where processing of other waste types, such as post-consumer packaging, deals with only a share of these difficulties—which already pose significant limitations to be overcome [16,17], CDW processing entails almost all central limiting factors for sensor technology simultaneously. What is more, multiple works and reports highlight the severe economic constraints that limit the development and testing of complex, state-of-the-art sensors that are suitable for implementation in rough environments and that can provide high-grade data at the same time [18,19,20]. For instance, Bayram et al. recently conducted a real case study to analyze the economical implications of advanced recycling of CDW using a life cycle costing approach and found that even with highly selective demolition, advanced recycling for the production of aggregates with higher quality is less cost-effective compared to conventional recycling [6]. This implicates a severe limitation for CDW processing companies to invest in advanced technologies for the production of high-quality recycled aggregates and thus also limits implementability of recyclable CDW material in high-grade recycling applications.

### 1.1. Relevance of Sensor-Based Quality Assurance in CDW Processing

Despite the aforementioned difficulties, innovations regarding automated quality monitoring and control in this area are important in reaching higher recycling rates for high-quality applications, such as building construction. More specifically, the large amount of yearly CDW combined with its high untapped potential—a recent study by the World Wide Fund for Nature describes a possible reduction of 60 megatons of CO_2_ equivalents, 66 megatons of resources, and one hectare of land use in Germany alone [21]—provides good reason for the development of automatization strategies for quality monitoring and control. What is more, a recent report on the potential and existing factors that limit sustainability in the construction sector identifies mandatory recycling rates and material specifications and digital infrastructure and transparency as two of eight main fields of action needed in order to create a sustainable transformation in the German construction sector [22]. To this end, automated quality monitoring and control systems would be an important part of the solution, as these systems could provide detailed information on the material specifications of the entire material stream being processed at a facility, could provide increased transparency with this information (for instance in the form of data about material quality in a digital product pass), may increase trust in using the secondary instead of a primary raw material, and could boost the development of an established digital infrastructure [19,22]. Similar conclusions are also drawn Europe-wide by the European Commission [23]. Likewise, given decreasing trends in road construction demands in Germany, where recycled aggregates are relatively often used, together with an increase in building constructions that are reaching the end of their life, it is now even more crucial to reduce environmental impacts by substitution of primary components with RC aggregates [24,25,26,27,28,29,30,31]. This can only be performed with high-quality aggregates, for which sensor-based quality assurance appears to be an important technology in increasing transparency regarding the true quality of (semi-)processed material on the one hand and in improving the quality by automatic optimization of the process on the other [7]. Additionally, if more detailed information on the material’s quality were available, this could lead to a clearer distinction between higher- and lower-grade material prices or could even be the basis for dynamic pricing, which could incentivize the use of recycled material instead of primary aggregates [6,18].

### 1.2. Related Work

To the authors’ knowledge, to this day, no overview of existing sensor-based quality assurance technologies across multiple research and application areas, regarding both raw and post-consumer material streams, has been provided. For instance, ref. [32] focuses only on one type of sensor and a single material stream. A similar constraint is used by [33], where the focus is on hyperspectral imaging (HSI) sensors for anaerobic digestion. On the other hand, refs. [34,35] describe a multitude of sensors but restrict their work to food products, and the extensive review by Kroell et al. is constrained to only include optical sensors in the context of mechanical recycling [1]. So, research across the different research areas—regarding both primary and post-consumer chains of production—is scattered, which limits the ability for less advanced areas to adapt established technologies found in more advanced areas in order to foster efficient innovation [36,37].

### 1.3. Aim and Research Question

Since the current scatteredness of existing research regarding sensor-based quality assurance hinders less advanced areas in efficiently adapting and developing such technologies, this work aims to systematically map available research and technologies regarding sensor-based quality assurance. Additionally, the current state of research and potential automatization strategies are described in the context of construction and demolition waste processing, as this waste stream contains a combination of complexities that are only present in part in other waste streams. To do so, an additional goal of this work is to describe a clear and holistic definition of the different terms and synonyms around quality assurance in the various chain of production phases given that the current use of terms is inconsistent or limited to a single production process or material stream [1,38]. With the proposed definitions, a generalizable conceptualization of common terms is provided to remove ambiguity when using these terms. Similarly, as the focus is on automated analysis, the same should be undertaken for the terms relating to the time span of analysis, which are often applied with inconsistent definitions [39]. The main research question is therefore defined as follows: *What is the current state of research regarding sensor-based quality assurance technologies developed for automatic quality control and monitoring applications in construction and demolition waste processing compared to other application areas across the chain of production?*

In the following, in Section 2, first, the systematic reviewing method used is described, after which the central concepts are defined (Section 2.1) and the corresponding search strategy is specified (Section 2.2). In the last part of the Materials and Methods Section, the conducted classification procedure is detailed (Section 2.3), which forms the basis for the overview of the existing research and technologies presented in this work. Subsequently, in Section 3, the results of the search and selection procedure and conducted analyses on the final collection of works are described. Corresponding interpretations and conclusions are presented in the last chapter, Section 4.

## 2. Materials and Methods

To achieve the goal of systematically mapping existing research of inline and online sensor-based approaches for quality assurance in pre- and post-consumer chains of production in a holistic and generalizable manner, a scoping review was conducted. Scoping reviews can embed multiple goals, as described by [40]. That is, a scoping review is a suitable method when it is used for the following: (1) to map and evaluate the current state of knowledge on a predefined topic; (2) to clarify central concepts; (3) to investigate applied research methods with respect to a certain topic; (4) to identify the main characteristics related to a predefined concept; (5) to map imperative knowledge as a precursor to systematic reviews; or (6) to identify and analyze knowledge gaps. This indicates that scoping reviews are focused on answering the type of research questions that focus on the identification of characteristics, factors, or fundamental concepts by mapping, reporting, and evaluating these aspects [40]. Accordingly, this type of systematic review mainly differs from the traditional systematic literature review in the sense of a broadened scope [40]. To conduct the scoping review method in a valid way, the Preferred Reporting Items for Systematic reviews and Meta-Analyses extension for Scoping Reviews (PRISMA-ScR) was used as the methodological framework [41,42].

### 2.1. Definition of Central Concepts

**Figure 1:**
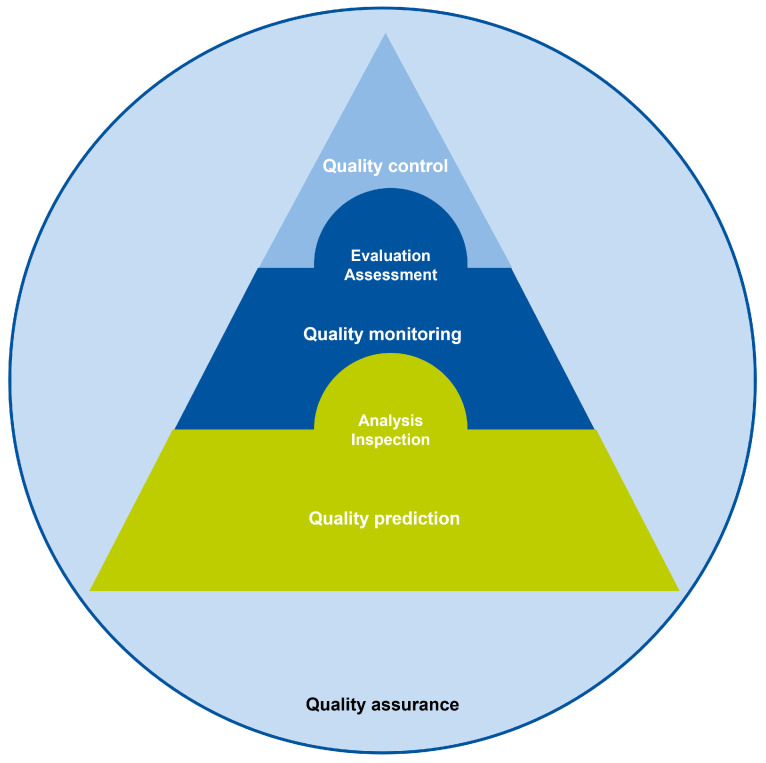
Conceptualization of the different quality-related terms.

Different related but by no means identical terms regarding aspects of quality assurance have been used interchangeably across the literature. As such, as part of the scope identification during the scoping review process, one of the goals was to provide a clear and generalizable conceptualization of frequently used terms and remove ambiguity when implementing these terms. The term quality assurance is appointed as the overarching concept, embedding all other quality-related terms as used in the different production-process-related research works. Herein, quality assurance is defined as *actions taken to ensure products are as good as they should be.* Within quality assurance, quality monitoring, quality control, and quality prediction or estimation take place, which intersect and whose definitions are based—albeit generalized to fit a broader scope—on [1]: embedded in both quality monitoring and quality control is the concept of quality evaluation or assessment, whereas quality analysis or inspection is at the core of all three subsets of quality assurance. Quality monitoring is defined as the passive real-time supervision of product quality, where usually some quality norm is present to which the acquired data are compared. For instance, a monitoring system can accumulate the share of contaminants detected in a waste processing stream to give an indication of the quality of that material stream, which in turn can be compared (either by the system or manually) to required purity levels. Conversely, quality control is defined as the active supervision and regulation of product quality. That is, where a quality monitoring system merely oversees what the quality of a product is, quality control systems use these findings to actively adjust the product and its quality. An example of quality control is the control of a printing head during additive manufacturing based on occurrences of over- or underfilling in the object being printed to optimize the object’s quality. Finally, quality prediction or estimation is the sole act of using available sensor information to make assumptions on the respective product’s quality. This especially regards using collected data for (offline) model training, for instance, to assess if a certain model is suitable for predicting a particular quality indicator or making quality predictions into the future. The latter is, for example, frequently used in waste water treatment plants to forecast influent and effluent parameters of the water. Using these conceptualizations, the three main quality assessment subcategories can be hierarchically ordered: quality control is positioned at the highest level, as this requires both quality monitoring and quality prediction when implemented in practice. A monitoring system, in turn, also requires the prediction of a product’s quality based on the analyzed quality indicator. Quality prediction regards some kind of forecast or prognosis on the quality of a product, without comparing this value to a defined norm. Lastly, where quality evaluation or assessment explicitly judges the available evidence, quality analysis or inspection only regards an examination or determination of the product’s quality without comparing it to a specified standard. An overview of the different concepts and their overlap can be seen in Figure 1.

Moreover, another set of terms that come with different definitions comprises concepts related to the time span of analysis: inline, online, atline, and offline. A summary of the differences between these concepts on which the definitions are based is presented in Table 1. Following the definition by Kessler, inline and online differ from atline and offline in the time in which the respective information is obtained. That is, inline and online approaches have information acquisition rates that are faster than the time in which process or material properties change [39,43]. Consequently, offline analysis is defined as sampling and transport to a device, usually in a central laboratory, for measurement. Atline, on the other hand, regards either manual or (semi-)automatic sampling and measurement with a device near the production process. Online analysis, however, allows continuous correlation between acquired information and process or product characteristics. With online analysis, usually, a bypass is used in which the bypassed sample is measured instantly and the measurements are acquired, evaluated, and sent to a central system (e.g., a central computer or process control system). The time this takes should, however, always be shorter than the time it takes for the product characteristics to change. The main difference between online and inline analysis is that where online analysis contains a bypass, an inline analysis system is directly integrated in the product stream, allowing instant information acquisition on product characteristics in the product or production process stream. According to Kessler, inline analysis can also be replaced with the term in situ analysis [39].

Important to note is, however, that the definitions described by Kessler have been developed in the context of chemical analysis, which neglects the nuance in time duration between sensors collecting information and the use of a computational model to perform the actual analysis. For instance, a sensor can be placed directly in the process stream, taking RGB images of the complete stream and sending these to a central computer where the data are subsequently stored in a database. The sensor as such operates inline. The collected data may, however, be implemented in a data processing and analysis pipeline months after the material passed by the sensor. Depending on the interpretation of “obtained information”, this could be either in- or online or atline—or even offline, if the analysis is conducted far from the process stream. To clear this ambiguity, we define approaches according to the gathering of the (raw) data using the sensors and potential for direct analysis. That is, if a work develops a lab-scale version of a particular (production) process and uses sensors to directly collect data from this process stream in order to develop a model for subsequent analysis—which could in turn straightforwardly be implemented to instantly output analysis results when added to the sensor-based data acquisition pipeline, it is considered in- or online.

**Figure 2:**
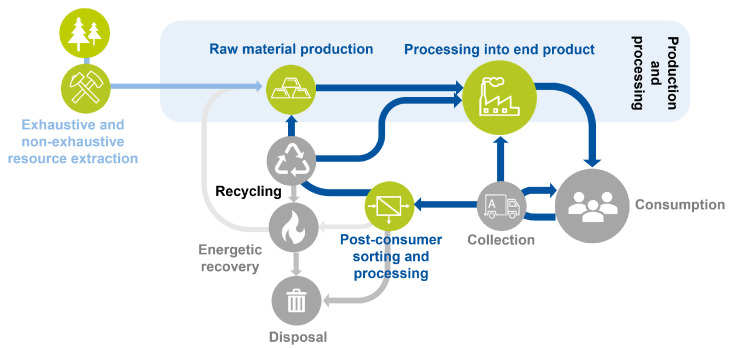
Adapted framework chain of production phases used by the Department of Anthropogenic Material Cycles (e.g., [46,47]). Green: considered; gray: out-of-scope.

A third conceptualization required for allowing a clear definition of the scope regards the chain of production. Theory on production chains is generally not optimized for both primary and secondary (raw) material streams [44,45]; however, product streams with a mix of primary and secondary raw materials are becoming more established [45]. In recent years, the Department of Anthropogenic Material Cycles developed a general model describing the different phases from raw material extraction to consumption and subsequent recycling back into production. Depending on the focus, this model can be specified to a single material stream but can also be used to present the different R-strategies or certain perspectives, such as the waste management or production perspective. The model is, for instance, used in [46] as a basis for the assessment of metal life cycles and was most recently presented on the *Berliner Konferenz Metallkreisläufe* [Berlin Conference for Metal Life Cycles] [47]. For this work, the model is slightly adapted: first, an additional division into exhaustive and non-exhaustive raw material extraction is made, where exhaustive refers to non-renewable resource removal from the earth and non-exhaustive to extraction of renewable sources from the earth, like farming. Second, raw material production and processing into end product are clustered into the overarching concept production and processing, as the focus is not so much on the specific steps of pre-consumer production and processing but on the division between pre- and post-consumer phases. The adapted version of this framework can be found in Figure 2, where the green icons are the phases taken into account in this work. Accordingly, the gray icons refer to the parts that were not considered, mainly because the implementation of (semi-)static sensor technology for quality assurance is not directly applicable in these phases.

Finally, to categorize the works according to technological maturity of the described system, the standardized technology readiness level (TRL) framework from the European Commission is used. In this framework, nine phases are defined, ranging from a technology at the start of its development (1) to a technology being commercially ready (9). Levels 1 to 3 are sometimes described as the discovery phase, levels 4 to 6 as the subsequent development phase, levels 7 and 8 as the demonstration phase, and the last—level 9—as the deployment phase. More specifically, level 1 covers fundamental research regarding basic principles, level 2 focuses on the formulation of the technological concept and level 3 refers to the existence of an experimental proof of concept. Then, level 4 regards successful technological validation in the lab, whereas levels 5 and 6 regard validation and demonstration in a relevant (industrial) environment, respectively. TRL 7 is the subsequent system prototype demonstration in the full-scale operational environment, and TRL 8 means the system is complete and qualified for deployment. As previously described, level 9 regards commercial deployment [48,49].

### 2.2. Search Strategy

As the scope of this review is automated quality assurance, only inline and online (sensor-based) analysis approaches are considered. For the same reason, as indicated in Section 2.1 and in Figure 2, the scope is constrained to only include resource extraction, raw material production, raw material processing into end products, and post-consumer sorting and processing. A further constraint is defined for the type of sensors: in this work, only wired sensors are considered, as the complexity of quality assurance problems—especially in the case of (construction and demolition) waste processing—requires stable, robust, and well-performing sensor technology, which are aspects that are often not yet met by wireless sensor applications [50,51]. Moreover, as automatization technology is evolving rapidly [52], it was decided to focus on works from the past ten years, excluding all works published before 2014. Furthermore, to allow in-depth examination of the applied technologies and procedures, only book chapters and journal articles were included, thus excluding reviews and conference publications due to their general lack of depth when it comes to describing technological implementation. Additionally, many of the findings presented for conferences are later published in journal articles in an extended format with the desired level of specificity.

Considering the lack of constraints on research and application area, the search process was conducted as an iteratively optimized sequence of systematic steps based on the intermediate findings. A first, intentionally broad search was conducted on three databases: Scopus for its large repository, ACM for further specification of computer-science-related works, and PubMed for further specification of works in the medical and biological domains. For the second search, the same three databases were used for the same reasons. In this search, additional focus was put on the waste management context, as this is the perspective from which this work has been written. Moreover, this search query is closely related to one of the search queries used by [1]. This is undertaken for two reasons: (1) to provide an updated collection, and (2) to examine if a verified and specific search leads to relevant works not found by the previous, more general search. Due to most publications already being present in Scopus, for the third search, it was decided to use Scopus only. This last search was based on the results and experiences from the first two searches and aimed to include works previously excluded by combining the broadness of the first search with the specificity of the second search. The first search was conducted on 25 March 2024, the second on 1 April 2024, and the last on 30 June 2024. In Table 2, the three search queries as applied in Scopus are presented.

During the filtering process, mainly because of the wide scope in terms of research and application areas, a conservative approach was used, where each step was conducted two or three times, filtering out only those works that clearly did not meet the requirements. This allowed us to get to know the various concepts, perspectives, and implications from works across the different areas and therewith improve the judgment of a work’s relevance.

More specifically, the following steps were taken to acquire the final body of works analyzed in this review. First, a general cleaning procedure was conducted to remove duplicates across the three databases and to remove works that do not meet the general requirements: being either a journal article or book chapter and being published after 2013. Moreover, retracted items were also removed in this step. Next, each work was filtered based on its title. That is, works where removed if the title met one or more the following exclusion criteria: (1) clear indication of non-sensor-based research (e.g., works investigating sensory responses of humans); (2) clear indication that the work does not regard one of the four considered phases of the chain of production (e.g., home monitoring); (3) clear indication that the work does not cover quality assurance technology in itself (e.g., works that investigate optimal positioning of wireless sensor networks); (4) clear indication that the work covers wireless sensing only and does not use wired sensors.

A similar set of criteria was subsequently used for the abstract screening process. Here, the criteria were extended to also exclude works that have a clear offline or atline analysis procedure and have no explicit goal of developing an inline or online quality assurance system.

For the content screening, first, all works meeting one or more of the aforementioned exclusion criteria were discarded. Then, a more in-depth analysis of the described research was conducted, discarding all works that do not meet the defined inclusion criteria: (1) at least one (wired) sensor that records one or multiple aspects of the final product, final product components, or input material is described; (2) the sensor should be recording these aspects in an inline or online manner; (3) the goal of developing an inline or online quality monitoring or quality control system is explicitly mentioned and is a viable subsequent step.

### 2.3. Classification Procedure

After collecting the final body of works, each work underwent in-depth analysis. The findings were documented and organized for subsequent analysis. More specifically, next to the general reference information, the sensor type, TRL, use of process sensors, use of multiple sensors, use of AI, the covered quality assurance part, the research areas (as defined by Scopus), the application area, the phase in the chain of production (based on the model presented in Figure 2), the assessed quality indicators, and whether or not the sensor was self-made were organized in a tabular format. The sensor type describes the general sensor category, such as “VIS” or “temperature and thermography”. This categorization was determined by iteratively defining and clustering the identified categories until a reasonable number of categories with sufficient difference between categories was established. In this work, less focus is on the technological functioning of the different sensor types. As such, in Table 3, an overview of recommended works for each of the main sensor types is provided.

By using the TRL framework from the European Commission as described in Section 2.1, it is possible to standardize the categorization of the technology implementation level across research and application areas, which eases subsequent comparison. Here, quality prediction implementations are always considered to be in the lower range of TRL—from TRL 2 up to TRL 5, depending on how the validation is conducted (e.g., in- or online (higher TRL) or offline (lower TRL) and in a pilot plant (higher TRL) or with a prerecorded laboratory data set (lower TRL))—as the overarching aim of each included work is the development of an automatic quality monitoring or quality control system. Consequently, quality monitoring and quality control implementations have a minimal TRL of 4, as the in- or online approach of the entire system, including raw data processing into an output describing one or more quality factors, is obligatory. Works that describe such applications can be labeled with a maximum TRL of 9, again depending on the implementation environment.

**Figure 3:**
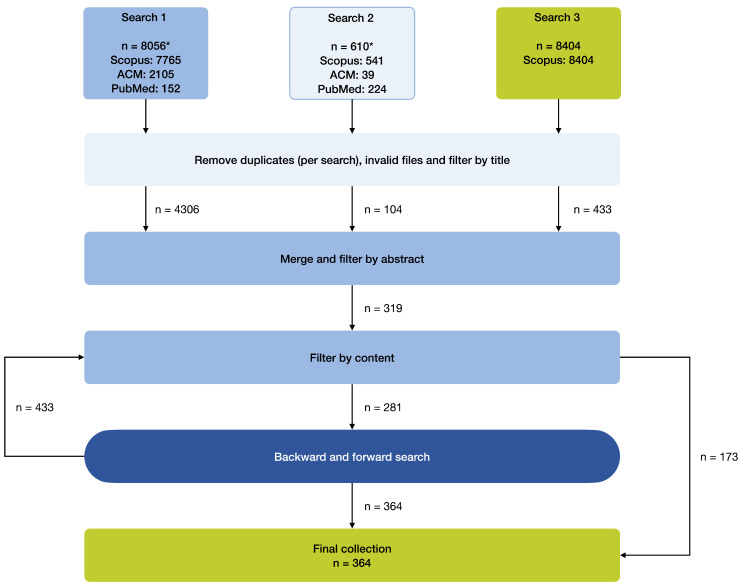
Resulting number of works for each step in the conducted selection procedure. * The number of unique items retrieved from the three databases combined.

Next, the *use of process sensors* and the *use of multiple sensors* aim to give more context to the exact implementation: the use of process sensors describes in a binary format (i.e., “yes” or “no”) whether one or more process sensors, such as for measuring the RPM of a motorized machine, were used in addition to the material quality assurance sensor(s). Similarly, the use of multiple sensors describes whether or not more than one sensor was used as part of the quality assurance system. The additional sensors can be both process or material quality assurance sensors. However, the variable *assessed quality indicator* only regards those quality indicators that are directly or indirectly measured by the material quality assurance sensors. This distinction is made to make clear which sensors have were for quality assurance applications without losing the information on the setup of the entire system. The *use of AI* and *self-made sensor* as the last two binary variables indicate whether some form of AI—such as a deep learning model for quality prediction—or a self-constructed sensor were implemented, respectively. The covered quality assurance part categorizes the works in either “quality prediction”, “quality monitoring”, or “quality control”, where the highest achieved assurance category (see Figure 1) is the assigned label. Lastly, the application area describes the more practical application context, as opposed to the research areas extracted from Scopus. The categories in *application area* were iteratively defined by identifying each exact application and then clustering these into 16 categories. An overview of all categories can be found in the Section 3, for instance, in Table 4. The search process, including the resulting number of works, are visualized in Figure 3. Further details on the review procedure can be found in the corresponding protocol, which was published on the Open Science Framework platform [91]. The full overview of works with according categorizations is provided in the Supplementary Materials.

## 3. Results

**Figure 4:**
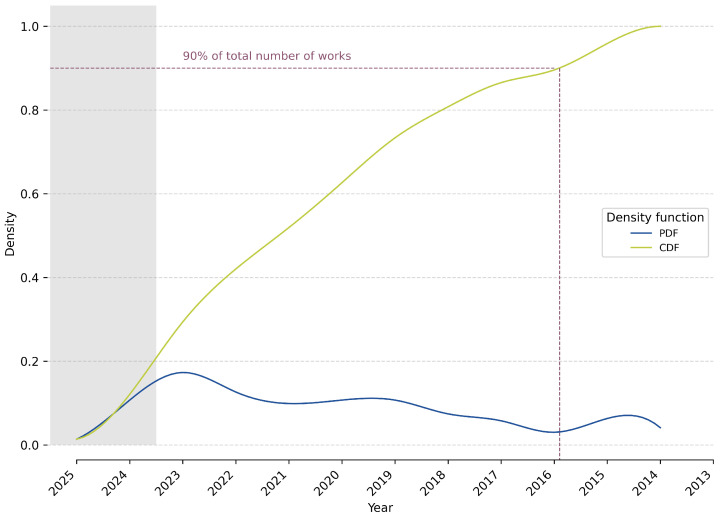
Cumulative (CDF) and probability density functions (PDF) of works over time, indicating that 90% of the works are from 2016 or later.

The aforementioned search and selection procedure resulted in a final collection that consists of 364 works. In total, 90% of these works were published in 2016 or later (see Figure 4). The works cover 21 different research areas in 72 different constellations. The most frequent research areas are *engineering* (243 works), *physics and astronomy* (124 works), and *computer science* (109 works). However, there are only 20, 7, and 1 work(s), respectively, that exclusively apply to one of these research areas. Additionally, 293 works have multiple research areas or the research area *multidisciplinary* assigned. An overview of all sources, organized per research area, can be found in Table 5. Note that one work can occur in multiple rows, indicating that it applies to multiple research areas. The main goal of this work is to identify and organize the different types of sensor technologies used for quality control and quality monitoring systems across the different application areas, research areas, and TRLs. To allow the distinguishing of certain trends, it is first important to identify patterns regarding the quality assurance parts, TRLs, and production process parts.

**Table 4:** Distribution of works across TRL, year, phase in chain of production, and application area.

TRL	%	Year	%	Phase in the Chain of Production	%	Application Area	%
2	2.5%	2014	4.1%	Exhaustive resource extraction	2.7%	Additive manufacturing	10.2%
3	69.8%	2015	6.3%	Non-exhaustive resource extraction	11.0%	Ceramics manufacturing	1.4%
4	15.7%	2016	30.2%	Production and processing	76.9%	Concrete and cement production	3.3%
5	5.8%	2017	5.8%	Post-consumer sorting and processing	9.3%	Electronics manufacturing	6.6%
6	3.3%	2018	7.4%	-	-	Food production and harvesting	15.7%
7	2.5%	2019	10.7%	-	-	Food storage	4.9%
8	0.3%	2020	10.7%	-	-	Fuel and energy production	0.8%
9	0.3%	2021	9.9%	-	-	Glass manufacturing	3.0%
-	-	2022	12.6%	-	-	Metals manufacturing	12.9%
-	-	2023	17.3%	-	-	Mining	2.5%
-	-	2024	1.4%	-	-	Miscellaneous	4.9%
-	-	2025	1.4%	-	-	Pharmaceutics and biomanufacturing	5.8%
-	-	-	-	-	-	Plastics manufacturing	8.0%
-	-	-	-	-	-	Recycling	9.3%
-	-	-	-	-	-	Unspecified	1.4%
-	-	-	-	-	-	Welding	9.3%

**Figure 5:**
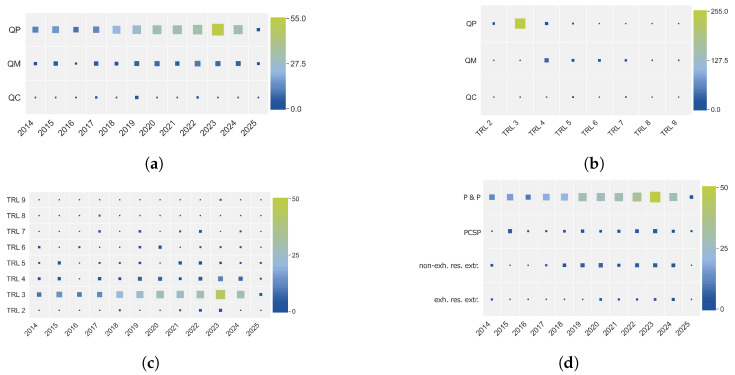
Heat maps visualizing co-occurrences of different aspects, where both size and color indicate frequency in terms of the number of sources. A bigger size of the square and a green color indicate a high number of sources. (**a**) Heat map of the number of sources per assurance type (QP: quality prediction; QM: quality monitoring; QC: quality control) and year. (**b**) Heat map of the number of sources per assurance type (QP: quality prediction; QM: quality monitoring; QC: quality control) and technology readiness level (TRL). (**c**) Heat map of the number of sources per technology readiness level (TRL) and year. (**d**) Heat map of the number of sources per phase in the chain of production (P & P: production and processing; PCSP: post-consumer sorting and processing; non-exh. res. extr.: non-exhaustive resource extraction; exh. res extr.: exhaustive resource extraction) and year.

In Figure 5a,c,d, it can be seen that quality prediction (assurance part), TRL 3 (i.e., experimental proof of concept), and production and processing (phase in the chain of production) occur most frequently and all reach their highest point in 2023. Moreover, even though it was expected that there would be an increase in TRL for the more recent publications, this trend is not distinguishable in Figure 5c. Additionally, where quality prediction mainly regards TRL 3, quality monitoring is more spread out across TRL 4 to 7 (see Figure 5b), which coincides with the given concept that a quality monitoring system embeds enhanced complexity and requires real-time testing in a representative environment.

**Figure 6:**
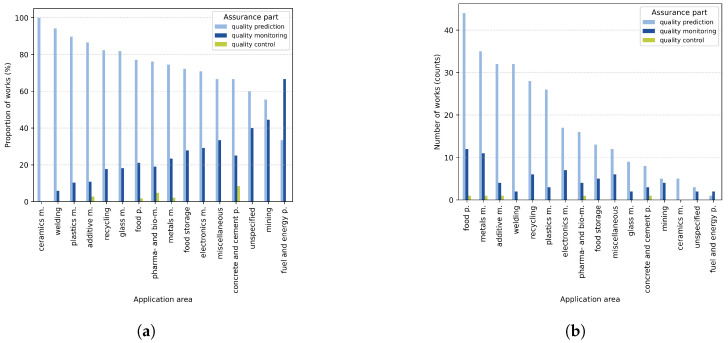
Bar chart of the relative and absolute distributions of works across application areas, categorized by assurance part. The abbreviations “m.” and “p.” refer to manufacturing and production, respectively. (**a**) Proportion of works per assurance part for each of the identified application areas. (**b**) Number of works per assurance part for each of the identified application areas.

In terms of presence across application areas, quality monitoring is relatively (i.e., by proportion) most frequent in fuel and energy production application areas (see Figure 6a). However, this includes merely two sources or 0.8% of the total body of works (see Table 4). Similarly, mining has relatively many publications that regard quality monitoring but covers only 2.5% of the total body of works altogether. When it comes to the absolute number of sources, food production and harvesting contains the most quality monitoring- and prediction-related publications, which is visualized in Figure 6b. What is more, Table 4 shows that this application area contains the highest number of works overall, covering almost 16% of the complete body of works used in this scoping review. The second-largest application area, metals manufacturing, also has the second-largest number of publications regarding quality monitoring. However, this is not true for additive manufacturing, where only 4 of its 37 works regard quality monitoring. Interestingly, the three most prominent application areas also contain a publication that considers quality control.

### 3.1. Sensor Technology

**Figure 7:**
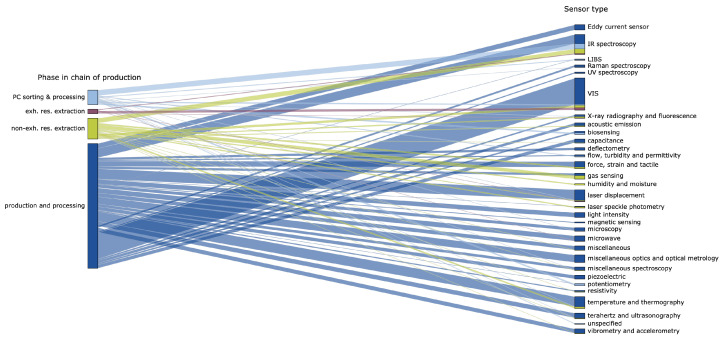
Parallel categories plot where the connections in terms of phase in the chain of production (PC: post-consumer; exh. res.: exhaustive resource; non-exh.res.: non-exhaustive resource) and sensor type for each work are visualized. The size of each bar is based on the number of works, whereas the color is added to distinguish between the different phases in the chain of production.

After clustering, 31 different sensor types were distinguished, where VIS or visual imaging (mostly industrial CCD or CMOS color cameras) and infrared spectroscopy (mostly near-infrared sensors) are most prominent. However, laser displacement technology like 3D laser triangulation or profiling and thermography are also relatively frequently used. That is, the former is used in 23 different works and the latter in 12 works, compared to 66 and 35 uses of VIS sensors and infrared spectroscopy, respectively. Where visual imaging sensors are relatively often used in the context of welding and additive manufacturing, infrared spectroscopy is a popular technology in both recycling and food production and harvesting. That is, well over 21% of the works regarding welding use VIS sensors. Similarly, for additive manufacturing applications, this value is almost 18%. When it comes to infrared spectroscopy, this technology is used in over 34% of the food production and harvesting works and nearly 27% in the context of recycling. Moreover, in Figure 7, it can be seen that some sensors, like Eddy current sensors, magnetism-based sensors, light intensity sensors, and piezoelectric sensors, are only used in production and processing applications.

Furthermore, there are 117 works that implement multiple sensors. Most of these works regard food production (57), metals manufacturing (47), and additive manufacturing (37). More specifically, however, there are eight works where sensor fusion is explicitly mentioned in the title, of which three are from a welding context. The remaining works describe additive manufacturing applications (also three), food storage (one) or metals manufacturing (one). For instance, Lee et al. use average current, average voltage, and mixed gas flow data as well as RGB image data for determination of the welding bead quality. Besides higher performance, adding non-VIS-sensor data to complement the acquired images also expands the information that can be extracted, allowing a more detailed weld bead inspection, which was not possible when using these data separately [214].

### 3.2. Quality Control

There are five works that implement a quality control system, three of which achieve TRL 5. The remaining works achieve TRL 7. Most of these five works are performed in the context of some form of manufacturing, including metals manufacturing [277], additive manufacturing [278], and biomanufacturing [445]. One work regards concrete and cement production [173] and is detailed in Section 3.3, and another describes a system for timber harvesting, which is categorized under food production and harvesting [96]. The distribution of quality control works is visualized in Figure 6.

Liu et al. use microscopy for defect detection during the additive manufacturing process. More specifically, they aim to detect microscale filling defects like under- and overfilling. This information is then forwarded to the control system linked to the cooler in the printing head for parameter optimization and thus optimization of the printed object quality [278]. They validate this system for one type of filament on a laboratory scale, printing a limited set of test objects.

A different type of quality control system is implemented by Mikkelstrup et al., where a welding robot is adjusted to scan the weld before and after quality optimization treatment of the weld seam [277]. That is, a welded seam is analyzed for its quality, after which it is treated accordingly and finally analyzed again in terms of quality. This is all performed by the system implemented in the welding robot. A laser line scanner based on laser triangulation generates a point cloud of the locations of the weld toes and corresponding grooves, as these are indicative of a weld’s quality. Even though an industrial welding robot is used, the system is verified on merely one type of material and one pair of plates welded together, creating 25 straight seams of 50 mm per seam for verification.

In [445], a continuous blending and tableting process setup is developed on a laboratory scale to investigate the use of Raman probes for active pharmaceutical ingredient (API) control, in this case caffeine concentration by adjustment of the feeder speed. That is, one Raman probe is placed above the conveyor belt transporting the blended material to the tableting machine and one probe is placed directly after the tableting machine. Both probes provide real-time spectra from which both the caffeine concentration and speed for the (caffeine) feeder are calculated. In total, 39 calibration samples with different concentrations and constituents are used to gather baseline spectral data. The control system is verified by utilizing the known caffeine contents and comparing the Raman data with an offline reference method.

The work by Sandak et al. implements and tests a quality control system on a timber processor head to analyze a variety of quality factors of timber logs and use this information for subsequent processing steps like cutting instructions [96]. In this system, infrared spectroscopy sensors, vibrometry sensors, and laser displacement sensors work together to acquire four main quality factors. That is, using these sensors, defects and decay are detected (spectroscopy), the stiffness (vibrometry) is estimated, and branches are located (laser displacement). The authors describe difficulties in validating the system on an industrial scale, mainly due to the lack of reference data.

### 3.3. State of Research in CDW Processing Compared to Mining and Concrete Production

To determine the current state of research regarding sensor-based quality monitoring and control in CDW processing and identify potential for further research on acquiring reliable and detailed data on material quality in this sector, a more in-depth analysis was conducted. These results will be highlighted in the following. Moreover, to set the results into context, the works with application areas in mining and concrete production are used as comparison. This also allows for creating an overview specifically for production with mineral fractions in the context of building construction. Within the category “recycling”, 13 works are written in the context of CDW processing for the purpose of recycling. Additionally, 12 works apply to “concrete and cement production” and 9 works to “mining”. From this subset of 34 works, only three works achieve a TRL of 7, which is the highest TRL present in this subset. Most of the works present quality assurance on TRL 3 (19 works) and TRL 4 (10 works). Similarly, most of the works regard only quality prediction (25 works), and only one work implements quality control.

The works that achieve TRL 5 or higher use a variety of sensors—in most (60%) of the cases, in combination with AI. Visual sensors are used in two works, whereas laser-induced breakdown spectroscopy (LIBS), laser displacement, resistance, and terahertz sensors are used only once. The two works by Zhang et al. achieve the highest TRL and describe a mining application [193,449]. More specifically, the proposed systems aim to replace manual grade titration by using a combination of VIS sensors and AI for the prediction of tailings grade in the iron ore reverse flotation process. In both works, the system is validated in an industrial concentrator with four production lines. However, first, images are acquired to create an offline data set for training the prediction model. These images are acquired by placing an industrial area scan camera above the flotation tank. After completing the entire quality monitoring system, this system is verified by comparing the results of manual sampling with the live predicted tailings grade. This is performed using uniform sampling of four samples per day—of which two are taken in the morning and two in the afternoon—for four weeks, creating 80 data points for verification. A total of 25 additional data points for verification are created for nighttime production, as the focus in these works is to show the validity of the system across a variety of light conditions. Even though more details, like the time of year, origins of the material, and duration and acquired weight of the sampling, are missing, the images provided in these two works—especially in [193], where each class of froth grade is visualized—show high homogeneity in the material when it comes to, e.g., color and texture.

Even though the work by Yoon et al. achieves the same TRL, the proposed approach and application context differ [173]. That is, in this work ultrasonic sensors are used to estimate compressive strength of concrete towers during and after construction and move the mold to the next point at the right time. More importantly, in this work, the quality monitoring part of the sensor system is first verified on a lab scale after which the quality control system is implemented for several full-scale concrete tower construction processes under different temperature conditions. Additionally, explicit bounds for the quality variable are stated, which is lacking in [449]. In contrast, in [173], a clear description of the industrial validation in terms of quantitative data and true quality of the concrete towers is lacking.

Compared to the previously described works, the works by Chang et al. and Liu et al., who describe implementations for CDW recycling, operate on a lower TRL but are the most advanced within the CDW recycling context. Where Chang et al. focus on classifying the various materials and especially contaminants that occur in CDW streams using LIBS and a laser displacement sensor, Liu et al. present a system for classification of the proportion of attached mortar using infrared spectroscopy [237,381]. So, even though both works apply some form of contaminant detection, the system types are different. In both works, offline data sets for training of the AI model are created; however, ref. [381] only describes results of (offline) validation on the spectral data acquired with a laboratory scale setup. Ref. [237], on the other hand, also implements live validation in an industrially relevant albeit simplified environment compared to true CDW processing circumstances. In both cases, validation is conducted by pre-labeling sample material so that the true class is known. Notably, ref. [237] uses some form of sensor fusion, where the laser displacement sensor localizes the particles so that the LIBS laser beam is emitted onto the right location (i.e., on a particle) instead of the beam ending up between particles. Besides this work, two more works that use multiple sensors were identified in the context of CDW processing, but neither actively fuse the sensor data to acquire more information than the sensors could provide separately [310,421].

In [237,381], besides the relative high TRL, the normally complex CDW processing environments are simplified in multiple ways. Most importantly, both works use a limited particle size range (10–20 mm [381] and 0–16 mm [237]) despite wider ranges being common in practice. Even though the work by Chang et al. approaches the true complexities of the CDW processing environment with occluded particles and the presence of fine fractions, the stability required for the LIBS and laser displacement sensor make it only suitable for second-stage processing after comminution, using a conveyor system that is detached from the comminution and sieving machines.

When comparing works in the context of CDW processing with those describing applications in mining, VIS and X-ray sensors have thus far only been implemented on low TRLs. Infrared spectroscopy is, however, popular for both mining and CDW recycling across the entire range of TRLs and is also occasionally implemented in the context of concrete and cement production. Especially regarding VIS sensors, works from other fields utilize large (synthetic) data sets (e.g., [151,377]) to bridge the gap between simplified laboratory data and the complex material streams in real operation environments. Additionally, for instance, Bilodeau et al. describe problems that occurred with laser displacement sensing in the dusty environment of an open-pit mining operation and suggest potential solutions such as different sensor locations and methods to combat the dust [228].

## 4. Discussion

In this work, the current state of research regarding sensor-based quality assurance technologies for automatic quality control and monitoring applications across different phases of the chain of production is described. What is more, given the high potential of sensor-based quality assurance in solving current problems in the transition to a sustainable construction sector, works in this sector are compared to works in other application areas across the chain of production. As such, a scoping review was conducted to systematically map existing research in this context. Using a three-part selection procedure conducted at different time points in 2024, a final collection of 364 works was compiled. Here, it should be noted that the last search was conducted in June 2024, after which a backward search was conducted in November 2024, which means some of the most recent works—published at the end of 2024 and beginning of 2025—might be excluded in this.

Most of the works regard implementations of proof-of-concept quality prediction systems on a laboratory scale. With merely 5 out of 364 works describing a sensor-based quality control system implementation, the results suggest such technology in itself is still in its infancy. However, this interpretation should be nuanced in two ways. On the one hand, with higher technology readiness—which is inherently embedded in quality control systems—comes higher secrecy, as this usually means competitive advantage for the involved industrial parties. On the other hand, quality control is connected to and sometimes confused with process control. Even though in principle all quality control-related works were included with the defined inclusion criteria and such confusion is likely to be mitigated, the concept of process control in itself and such specific technologies were ignored. As such, process control systems that could be generalizable to quality control are to some extent left out.

When it comes to quality monitoring, as a preceding step to quality control system development, the application areas *fuel and energy production* and *mining* seem to be the most prominent by proportion. Looking at absolute numbers, however, *food production and harvesting* and *metals manufacturing* have the highest numbers of works. In general, the application areas that also have contributions regarding quality control are among the prominent areas for quality monitoring contributions, suggesting that these areas are the most advanced.

The types of sensors used for the investigated quality assurance systems generally vary for each work, but some form of spectroscopy is used in almost all works. Multiple factors could be the reason for the general popularity of spectroscopy, such as the type of quality factor investigated, the relatively wide applicability of spectroscopy technology, and the fact that it is generally inexpensive compared to alternative technologies. This tendency is also visible in the results regarding all works, where VIS and IR spectroscopy sensors are the most prominent. Moreover, the different types of sensors are generally used across a variety of application areas and TRLs. Additionally, sensor data are often combined with AI, suggesting that quality factors cannot be directly interpreted from existing sensor technologies and require subsequent processing.

Looking at construction and demolition waste processing specifically, the area seems to be less advanced, especially when it comes to the implementation of the popular VIS sensors on high TRLs. Furthermore, a comparison of works from the CDW processing, mining, and concrete production application areas shows the general lack of exhaustive descriptions on how the system is validated and why a particular experimental procedure is adequate. Similarly, some works explicitly describe difficulties in validating the developed quality assurance system in a relevant environment. Major research gaps thus seem to be the further upscaling of currently developed systems, and also the implementation and testing of other sensor technologies, such as terahertz sensors that can, for example, be used for material characterization or volume determination. Even though X-ray technology has potential in sorting out contaminants, given the generally high costs of this technology, the use of VIS and laser displacement sensing as an alternative is something CDW researchers and practitioners can focus on now, utilizing developments that have been made in other fields. More specifically, (semi-synthetic) data generation methods already used in other fields could be used to minimize sampling and labeling effort of the highly heterogeneous material and at the same time create larger, more informative and realistic data sets. This may bridge the gap that generally exists when implementing AI-based models in a real environment after offline training in a lab. Additionally, in line with [237], laser displacement as part of a multi-sensor system seems to be effective for operation environments close to real industrial CDW processing environments. As such, sensor fusion methods from, for instance, the field of welding, where such methods have been widely researched, will be a good starting point in advancing multi-sensor systems for CDW processing. However, it should be noted that welding and additive manufacturing generally operate in a static environment, whereas quality assurance systems for CDW processing operate in environments where the material is in a continuous flow, e.g., during transportation on a conveyor belt. This complicates calibration between the different sensors, which is also described in [237]. Besides required technical adaptations of existing technologies from other fields to make them operable in CDW processing applications, like dust and weather resistance, the economic aspect remains essential, as advanced recycling still brings limited to no economic value to CDW processing companies. This indicates that in general, technological innovations need to be inexpensive as they will otherwise not be invested in.

However, when such technologies are implemented in industrial environments, their detailed material quality data acquisition may become an important aspect in the development of, for instance, digital product passports, supporting the transition to a sustainable construction sector. So, to further and efficiently advance quality monitoring and control systems, an enhanced focus on the implementation on the higher technology readiness levels, development of cost-effective multi-sensor systems based on existing data fusion and data generation methods, and inclusion of detailed descriptions on how these systems have been verified will be beneficial, both within and beyond the context of CDW processing.

## 5. Conclusions

By systematically mapping the current state of research and highlighting the current limitations and existing potential for CDW processing as one of the more complex material streams for sensor-based system implementations, this work provides a novel basis for efficient adaptation of existing sensor-based quality assurance technologies and streamlined development of new technologies for the CDW processing sector. In this work, results show the relative infancy of sensor-based quality assurance developments in CDW processing compared to other application areas. However, it is argued that a focus on the development of (1) cost-effective, (2) multi-sensor systems based on (3) existing data fusion frameworks and (4) (synthetic) data generation methods that are in part already researched in other application areas, as well as (5) inclusion of detailed descriptions on the validation of these systems, can boost development of systems that are implementable in practice and support the transition to a sustainable construction sector.

## Figures and Tables

**Table 1:** Comparative overview of the concepts and definitions related to the time span of analysis.

	Inline/Online	Atline/Offline
Acquisition rate	Faster than change in process or material properties	Slower than change in process or material properties
Measurement location	In the material stream/in a bypass of the material stream	Near the production process/at a central location away from the production process
Sensor data processing	Processing and analysis happen directly or have potential to instantly output results	No potential for direct processing and analysis of sensor data

**Table 2:** The conducted search queries, based on the syntax used in Scopus.

Search Number	Search Query
1	TITLE (sensor AND quality OR monitoring) AND KEY (sensor AND quality OR monitoring) AND LANGUAGE (english) AND PUBYEAR > 2013 AND PUBYEAR < 2025 AND (LIMIT-TO (DOCTYPE,“ar”) OR LIMIT-TO (DOCTYPE,“ch”))
2	TITLE-ABS-KEY (quality AND (product OR assess* OR analys* OR control* OR monitor* OR assurance)) AND TITLE ((waste OR recyc* OR recover* OR “post-consumer” OR “postindustrial”) AND (sensor* OR *spectr* OR imag*)) AND PUBYEAR > 2013 AND PUBYEAR < 2025 AND (LIMIT-TO (DOCTYPE,“ar”) OR LIMIT-TO (DOCTYPE,“ch”) AND (LIMIT-TO (LANGUAGE,“English”))
3	TITLE-ABS-KEY (quality AND (product OR assess* OR analys* OR assurance OR spectr* OR imag*)) AND TITLE (sensor* AND NOT (“sensory” OR “sensorial”)) AND PUBYEAR > 2013 AND PUBYEAR < 2025 AND (LIMIT-TO (DOCTYPE,“ar”) OR LIMIT-TO (DOCTYPE,“re”) OR LIMIT-TO (DOCTYPE,“ch”)) AND (LIMIT-TO (LANGUAGE,“English”))

**Table 3:** Overview of the sensor-type categories and recommended sources explaining these technologies in more detail.

Sensor Category	Publications
VIS and optics	[53,54,55,56]
IR spectroscopy	[32,33,57,58,59]
Temperature and thermography	[60]
Laser displacement	[61]
Force, strain, and tactile	[62,63]
Gas sensing	[64]
Microwave	[65]
Terahertz and ultrasonography	[66,67]
Eddy current sensing	[68]
Vibrometry and accelerometry	[69,70]
Light intensity	[55]
Capacitance	[71]
Acoustic emission	[72]
X-ray radiography and fluorescence	[73,74]
Piezoelectric	[75]
Biosensing	[76,77]
Raman and UV spectroscopy	[78,79]
Deflectometry	[80,81]
Potentiometry	[82]
Flow, turbidity, and permittivity	[65,83,84]
Resistivity	[85,86]
LIBS	[87]
Humidity and moisture	[88,89]
Magnetic sensing	[90]

**Table 5:** Overview of the works, categorized by research area.

Research Area	Publications
Agricultural and biological sciences	[92,93,94,95,96,97,98,99,100,101,102,103,104,105,106,107,108,109,110,111,112,113,114,115,116,117,118,119,120,121,122,123,124,125,126,127,128,129,130,131,132,133,134,135,136,137,138,139,140,141]
Biochemistry, genetics, and molecular biology	[108,109,110,111,112,113,114,115,116,117,142,143,144,145,146,147,148,149,150,151,152,153,154,155,156,157,158,159,160,161,162,163,164,165,166,167,168,169,170,171,172,173,174,175,176,177,178,179,180,181,182,183,184,185,186,187,188,189,190,191,192]
Business, management, and accounting	[193,194,195,196,197,198,199,200]
Chemical engineering	[114,115,118,119,120,121,142,143,144,179,180,201,202,203,204,205,206,207,208,209,210,211,212,213,214,215,216,217,218,219,220]
Chemistry	[108,109,110,111,112,113,114,115,116,117,122,123,124,125,126,142,143,144,145,146,147,148,149,150,151,152,153,154,155,156,157,158,159,160,161,162,163,164,165,166,167,168,169,170,171,172,173,174,175,176,177,178,179,180,181,182,183,184,185,186,187,188,189,190,191,192,204,205,206,207,221,222,223,224,225,226,227,228,229,230,231,232,233,234]
Computer science	[127,128,129,130,131,132,145,146,147,148,149,150,151,152,153,154,155,156,157,158,159,160,161,162,163,181,182,183,184,185,186,187,208,209,210,211,212,213,214,215,235,236,237,238,239,240,241,242,243,244,245,246,247,248,249,250,251,252,253,254,255,256,257,258,259,260,261,262,263,264,265,266,267,268,269,270,271,272,273,274,275,276,277,278,279,280,281,282,283,284,285,286,287,288,289,290,291,292,293,294,295,296,297,298,299,300,301,302,303]
Decision sciences	[193,194,195,196,197,198,199,200,304]
Earth and planetary sciences	[216,228,305,306]
Economics, econometrics, and finance	[307,308,309,310,311]
Energy	[216,217]
Engineering	[114,115,118,119,120,121,133,134,135,136,137,142,143,144,145,146,147,148,149,150,151,152,153,154,155,156,157,158,159,160,161,162,163,168,169,170,171,172,173,179,180,181,182,183,184,185,186,187,193,194,195,196,197,198,199,200,201,202,203,204,205,206,207,208,209,210,211,212,213,214,215,216,217,218,219,220,228,229,236,237,238,239,240,241,242,243,244,245,246,247,248,249,250,251,252,253,254,255,256,257,258,259,260,261,262,263,264,265,266,267,268,269,270,271,272,273,274,275,276,277,278,279,280,281,282,283,284,285,286,287,288,289,290,291,292,293,294,295,296,297,298,299,303,306,312,313,314,315,316,317,318,319,320,321,322,323,324,325,326,327,328,329,330,331,332,333,334,335,336,337,338,339,340,341,342,343,344,345,346,347,348,349,350,351,352,353,354,355,356,357,358,359,360,361,362,363,364,365,366,367,368,369,370,371,372,373,374,375,376,377,378,379,380,381,382,383,384,385,386,387,388,389,390,391,392,393,394,395,396,397,398,399,400,401,402,403,404,405,406,407,408,409,410,411,412,413,414,415,416,417,418]
Environmental science	[116,121,126,138,174,175,179,188,189,190,205,217,307,308,309,310,311,332,333,334,419,420,421,422,423,424]
Health professions	[139,140,141]
Immunology and microbiology	[115,117,139,140,141,143,144,180]
Materials science	[142,206,207,208,209,210,211,212,213,214,215,219,220,229,230,231,281,282,283,284,285,286,287,300,334,335,336,337,338,339,340,341,342,343,344,345,346,347,348,349,350,351,352,353,354,355,356,357,358,359,360,361,362,363,364,365,366,367,368,369,370,371,372,373,374,424,425,426,427,428,429,430,431,432,433]
Mathematics	[232,233,288,289,290,291,292,293,294,295,296,301,375]
Medicine	[126,142,297,434]
Multidisciplinary	[302,435,436,437,438,439,440,441,442,443]
Pharmacology, toxicology, and pharmaceutics	[126,176,177,178,444,445,446,447,448]
Physics and astronomy	[142,145,146,147,148,149,150,151,152,153,154,155,156,157,158,159,160,161,162,163,164,165,166,167,168,169,170,171,172,173,181,182,183,184,185,186,187,191,208,209,210,211,212,213,214,215,229,234,298,299,356,357,358,359,360,361,362,363,364,365,366,367,368,369,370,371,372,373,374,375,376,377,378,379,380,381,382,383,384,385,386,387,388,389,390,391,392,393,394,395,396,397,398,399,400,401,402,403,404,405,406,407,408,409,410,411,412,413,414,415,416,418,430,431,432,433,434,449,450,451,452,453,454,455]
Social sciences	[116,139,140,141,417]

## Data Availability

The original contributions presented in this study are included in the article/Supplementary Materials. Further inquiries can be directed to the corresponding author.

## Supplementary Materials

The following supporting information can be downloaded at: https://www.mdpi.com/article/10.3390/s25144401/s1, Table S1: Final body of works with categorizations as csv file.

## Abbreviations

The following abbreviations are used in this manuscript:

HSI	Hyperspectral imaging
CDW	Construction and demolition waste
PRISMA-ScR	Preferred Reporting Items for Systematic reviews and
	Meta-Analyses extension for Scoping Reviews
RGB	Red–green–blue
ACM	Association for Computing Machinery
TRL	Technology readiness level
VIS	Visual imaging
IR	Infrared
UV	Ultraviolet
LIBS	Laser-induced breakdown spectroscopy
RPM	Revolutions per minute
AI	Artificial intelligence
CCD	Charge-coupled device
CMOS	Complementary metal oxide semiconductor
3D	Three-dimensional
CDF	Cumulative distribution function
PDF	Probability distribution function
P & P	Production and processing
PCSP	Post-consumer sorting and processing
PC	Post-consumer
non-exh. res. extr./extraction	Non-exhaustive resource (extraction)
exh. res. extr./extraction	Exhaustive resource (extraction)
m.	Manufacturing
p.	Production
